# The Association between Federal Nutrition Assistance Programs and Adolescent Food Security during the COVID-19 Pandemic: Evidence from Baltimore, Maryland

**DOI:** 10.3390/nu16172876

**Published:** 2024-08-28

**Authors:** Kristin Mmari, Kaitlyn Harper, Jon Kawatachi, Marina Jenkins, Susan Gross, Stacy Lu, Rebecca Skinner, Beth Marshall

**Affiliations:** 1 Department of Population, Family, and Reproductive Health, Johns Hopkins Bloomberg School of Public Health, Baltimore, MD 21205, USA; jkawata1@jhu.edu (J.K.); mjenki51@jhu.edu (M.J.); sgross3@jhu.edu (S.G.); slu47@jhmi.edu (S.L.); rskinne5@jhmi.edu (R.S.); bmarsha2@jhu.edu (B.M.); 2 Department of Environmental Health and Engineering, Johns Hopkins Bloomberg School of Public Health, Baltimore, MD 21205, USA; kharpe14@jhu.edu

**Keywords:** adolescent food security, COVID-19 food assistance, urban health

## Abstract

Given the lack of attention on adolescent food insecurity, the primary objective of this study was to assess the association of household participation in federal food assistance programs with food security status among adolescents in Baltimore during the COVID-19 pandemic. Adolescents, ages 14–19 years, were invited to participate in two online surveys. The baseline was implemented between October 2020 and January 2021, while the follow-up took place one year later from November 2021 to January 2022 after schools had re-opened. We then matched survey participants with household participation in food nutrition assistance programs using data obtained from the Maryland Department of Social Services. We used logistic regression to examine the association between food assistance program participation status and food insecurity. Additionally, to examine whether the impact of program participation on food insecurity changed between the baseline survey and one year later at follow-up when schools re-opened, a difference-in-differences analysis was conducted. The results showed no significant associations between adolescent food security and participation in any of the federal nutrition assistance programs. Increased attention on how best to improve adolescent food security in low-income households that can respond to the unique needs of adolescents is clearly warranted.

## 1. Introduction

Food insecurity is defined as having limited or uncertain access to nutritionally adequate and safe foods or having a limited or uncertain ability to obtain these foods in a socially acceptable manner [1]. In 2019, approximately 6.5% of United States (US) households with children under the age of 18 years experienced food insecurity; in 2020, the prevalence increased to 7.6% [2]. Data from the Census Bureau’s Household Pulse Survey, a weekly survey that started tracking health and economic outcomes during the COVID-19 pandemic, revealed a similar picture and showed that nearly one in three households with children reported that at least some household members sometimes or often did not have enough to eat over the preceding week [1]. Additionally, the burden of food insecurity is higher for Black, Indigenous, and other households of color due to historic and ongoing oppression resulting in wealth disparities. For example, in 2020, the prevalence of very low food insecurity for Black households with children was 13%, compared to 4.6% for White households with children [2]. These findings are concerning for several reasons. First, food insecurity is associated with poor health outcomes including diabetes, heart disease, asthma, anemia, depression, and mental health disorders [3,4,5,6,7]. Second, children’s exposure to adverse economic shocks, such as food insecurity, can have long-term health consequences that can be experienced for decades to come [8].

Evidence suggests, however, that not all children in a household are at equal risk of experiencing food insecurity. Research has shown that younger children are more often protected by adults from the effects of food insecurity compared to older children or adolescents [9]. An analysis of child-level food insecurity data from 2010 to 2011, for example, showed that households with adolescents were twice as likely to report some level of food insecurity and three times as likely to report very low child food insecurity compared to households with only younger children under five years of age [10]. This pattern is consistent with more recent studies showing a higher prevalence of food insecurity in households with adolescents compared to households with younger children [11]. Some have suggested that the higher rates of food insecurity among adolescents are because adolescents often have to reduce their own diet quality and quantity to protect younger children from experiencing food insecurity, while others have said that it may be more related to adolescents having a higher caloric intake need compared to younger children [10,12,13].

Food insecurity among adolescents is a critical problem to solve, irrespective of the reason, because it creates barriers to development that can have long-term negative health consequences well into adulthood. The period of adolescence is marked by rapid physical, emotional, and social development, which requires adequate nutrition intake to support this rapid rate of development. Adolescents who do not receive adequate nutrients and who experience food insecurity have been shown to be at higher risk for engaging in harmful behaviors, such as substance use, transactional sex, and shoplifting, which can further lead to mental health problems, negative reproductive health outcomes, and contact with the criminal justice system, all of which can further impact on their health and economic trajectories [14,15,16]. Yet, compared to younger children and adults, adolescents are far less studied in food security research.

To alleviate food insecurity and its consequences, federal nutrition assistance programs, such as the Supplemental Nutrition Assistance Program (SNAP), were developed by the United States Department of Agriculture (USDA) [17]. As the largest nutrition assistance program in the United States, SNAP provided benefits to approximately 35.7 million people living in 18 million households each month across the United States in 2019 [18]. During the COVID-19 pandemic, SNAP participation increased and provided assistance to over 40 million people each month in 2022 [19]. Prior to the pandemic, evidence on SNAP demonstrated primarily positive impacts among adults, such as improved food security and health outcomes [20,21,22,23]. Among children, however, the results were more mixed for food security. Several critics have said that SNAP benefits are often too low for households with children, which leaves many families who participate in SNAP still experiencing food insecurity [24]. In non-emergency times, the maximum household SNAP benefit is tied to the cost of the USDA’s Thrifty Food Plan, which is calculated as “the cost of groceries needed to provide a healthy, budget-conscious diet to a family of four” [25]. This plan, however, assumes that families purchase entirely low-cost raw materials and prepare all meals from scratch, which is often not feasible. Even during the last economic crisis, the Great Recession of 2008, one study found that additional SNAP benefits provided to families were not able to reduce food insecurity among children aged 2–18 years [26].

During the more recent COVID-19 pandemic, low-income families with children were faced with additional economic hardships due to increases in unemployment, rising food prices, and the closing of school and child-care centers that led to losing access to free and reduced-price meals [27]. In response, two key legislative actions were implemented by the federal government to specifically help families with children: (1) making changes to SNAP and (2) implementing a new program, called Pandemic Electronic Benefit Transfer (P-EBT). The changes to SNAP, associated with the Families First Coronavirus Act (FFCA), allowed increases in benefit allotments up to the maximum amount, expanded program eligibility, and waived or extended paperwork deadlines and interview requirements. In addition, the P-EBT program was provided to families to account for the loss of access to free or reduced-price school meals due to COVID-19-related school closures. Households were eligible to receive P-EBT if one or more children in the household received free or reduced-priced school meals or if they attended a school that offered free meals to all students. Households that received free or reduced-price meals, but did not have existing EBT cards, received a P-EBT card in the mail for each child in the household, with each child’s name on the card [28]. Indeed, in a recent study among households with children, P-EBT was shown to reduce food hardship [8].

In Baltimore City, city officials first became aware of the need to focus on adolescent food insecurity after a study conducted in 2018–19 revealed that more than half the adolescent sample experienced food insecurity and engaged in several harmful behaviors to obtain their own food [14]. As a result, the city decided to pilot the Summer SNAP program, a federal summer meals program, among existing SNAP families with adolescents in the household. The program provided an additional USD 30 per adolescent child (ages 14–17 years) per summer month onto existing EBT cards [29]. To select families for the pilot, city officials partnered with YouthWorks, a Baltimore City youth employment program, to randomly select adolescents who (a) completed an application to YouthWorks for the summer of 2020 and (b) lived in SNAP-eligible households (but were not necessarily enrolled in SNAP at the time). In December 2019, in anticipation of this pilot, city officials contacted the authors of this paper to design an evaluation of the impact of Summer SNAP on adolescent food insecurity. However, because of the pandemic and new changes to SNAP and the implementation of P-EBT, the design of the evaluation was changed to examine the associations of all three programs (the additional benefits with SNAP, P-EBT, and Summer SNAP) with adolescent food insecurity.

This paper presents the results of this evaluation study. To our knowledge, this is the first study that has focused specifically on the association of different federal nutrition assistance programs with the food security status of only adolescents in SNAP-eligible households.

## 2. Methods

### 2.1. Sample and Recruitment

Our sample for this study was recruited from a database provided by the Mayor’s Office of Employment and Development of 1100 adolescents, aged 14–19 years, who completed an application to YouthWorks, a Baltimore City youth employment program, and who lived in Baltimore City households eligible to receive SNAP benefits. The database contained contact information for recruitment and identification numbers needed to match participants to Maryland SNAP data. Invitations to participate in our study were sent to all adolescents in this database via the text messaging software Twilio [30] integrated with our data management software, REDCap version 14 (Research Electronic Data Capture) [31,32]. For adolescents under the age of 18, we first contacted caregivers via text message or phone to obtain permission to recruit their child. If caregivers provided permission, we contacted adolescents via text message to obtain assent. Adolescents 18 years and older were contacted directly by text or phone to obtain consent. Adolescents were considered a ‘non-responder’ if they failed to respond to three text or phone contacts.

### 2.2. Data Collection

Adolescents who agreed to participate were asked to complete an online survey at two different timepoints during the COVID-19 pandemic: baseline surveys occurred during the school lockdown period from October 2020 to January 2021, and follow-up surveys (post-lockdown) took place one year later, from November 2021 to January 2022, after schools had re-opened. To be eligible to participate in the follow-up surveys, adolescents had to complete a baseline survey to allow us to assess changes in food insecurity status. Each adolescent participant received a USD 25 gift card for each survey they completed. The survey included approximately 30 close-ended questions that asked questions about food security status, nutrition behaviors, and awareness about federal nutrition assistance programs. Survey data were collected and managed using REDCap electronic data capture tools hosted at the Bloomberg School of Public Health. REDCap is a secure, web-based software platform designed to support data capture for research studies [31,32].

To determine whether there was an association between adolescent food insecurity status and household participation in federal nutrition assistance programs, we matched household data from each adolescent survey respondent with data from the Maryland Department of Human Services using the Client Identification Number and the SNAP Program Assistant Unit Number through a data-sharing agreement. The state data included median monthly payments received by households from SNAP, Summer SNAP, and P-EBT at three different time periods: (1) pre-pandemic (1 December 2019 to 1 March 2020); (2) during the COVID-19 school lockdown (1 July 2020 to 30 September 2020), and (3) post-school-lockdown (1 February 2021 to 30 April 2021). Overall, 81.0% of adolescent survey participants were matched to the state administrative data. A chi-square test of independence showed that there was no significant association between adolescent food insecurity and being matched to the administrative database in the baseline survey, *X*^2^ (1, *n* = 284) = 0.16, *p* = 0.68, and the follow-up survey, *X*^2^ (1, *n* = 131) = 0.26, *p* = 0.61.

Ethical approval was obtained from the Johns Hopkins University Bloomberg School of Public Health Institutional Review Board (#12667).

### 2.3. Measures

*Demographic* measures, including age, household size, and job status, were obtained from survey responses. Age was dichotomized into two age groups: younger (<18 years) and older (>18 years) to reflect the differences in food insecurity that are often reported between younger and older children [10]. Household size was characterized as <4 members, 4 members, or >4 members, which aligns with USDA’s Thrifty Food Plan, in which households with less than four members receive slightly more SNAP benefits per person compared to a 4-person household, while households greater than four get slightly less per person [33]. Job status was categorized as having no job, a part-time job, or a full-time job.

*Food security status* was measured using the 9-item tool developed by Connell and colleagues in 2004, which was adapted from the U.S. Food Security Survey Module and has since been used in studies among adolescents in Baltimore [34]. Food security status was categorized into two groups based on the number of affirmative responses: food-secure (0–1) and food-insecure (2–9).

*Food assistance program participation* was categorized in two ways to understand whether adolescent food insecurity was associated with living in a household that participated in a federal food assistance program and/or with the particular amount a household received. First, we created dichotomous variables to represent participation in each of the three federal assistance programs (SNAP, P-EBT, and Summer SNAP). Household participation in federal nutrition assistance programs was ascertained if participants received the benefit in any of the months during the two time points based on the Maryland State Department of Human Services data. Additionally, payment amounts were summarized for each of the three relevant payment periods described above. The full baseline and follow-up surveys are included in the Supplementary Materials.

### 2.4. Statistical Analysis

Analysis of survey data was conducted using R version 4.2.1 (23 June 2022) [35] and Stata version 18.0 [36]. The median monthly benefit amount was calculated using the median monthly distribution amount during the three months corresponding to each time point (see Supplemental Table S1). A Shapiro–Wilk test was then performed and showed that the distribution of the overall median monthly payments departed significantly from normality at the time periods corresponding to the baseline (W = 0.90, *p*-value < 0.01) and follow-up surveys (W = 0.91, *p*-value < 0.01). Based on this, a non-parametric Wilcoxon rank sum test was used for comparison, and the median with interquartile range was used to summarize payments. To assess the relationship between food assistance participation status and food insecurity during the COVID-19 lockdown, participants were categorized into three groups: (1) those who received SNAP plus additional stipends from P-EBT and/or Summer SNAP, (2) those who received only the base SNAP payments, and (3) those who did not receive payments from any of the three programs during the lockdown time period but were eligible for SNAP. We used logistic regression to examine the association between food assistance program participation status and food insecurity during the COVID-19 lockdown. Additionally, to examine whether the association between program participation and adolescent food insecurity changed between the baseline survey during the COVID-19 lockdown and one year later at follow-up when schools re-opened, a difference-in-differences analysis was conducted to compare the change in risk of food insecurity between those who received P-EBT or Summer SNAP and those who did not, for the sample who completed both surveys. Household size, median monthly payment for the lockdown and post-lockdown periods, age group, and job status were considered as potential covariates for each regression model.

## 3. Results

### 3.1. Sample Characteristics by Food Security Status

**Figure 1:**
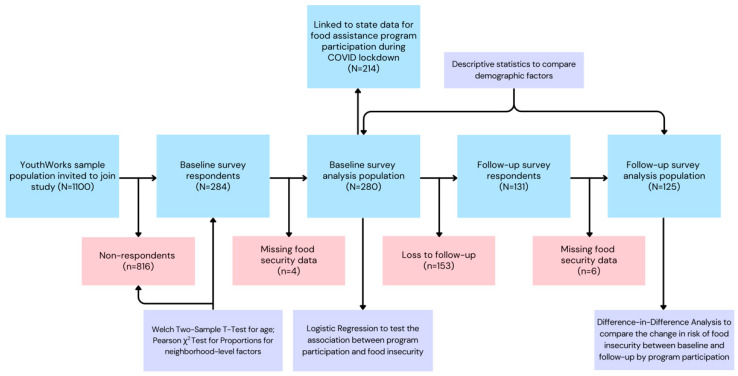
Schematic diagram describing the workflow of the study. Blue boxes represent sampling stages, red boxes represent exclusion, and purple boxes represent comparison tests.

A total of 284 adolescents participated in the baseline survey, of whom 131 participated in the follow-up survey (retention rate: 46%). Adolescents who completed the baseline survey were compared with adolescents who did not (*n* = 816) using a Welch two-sample *t*-test on age and neighborhood-level characteristics based on addresses (% of children living below poverty line, % of families receiving TANF, % of families receiving SNAP, and unemployment rate). No significant differences were observed, with the exception of age, in which adolescents who completed the baseline survey were significantly older compared to those who did not, *p* < 0.05). A small number of individuals were also excluded from analyses due to missing food insecurity data (baseline: *n* = 4; follow-up: *n* = 6). Of the 280 adolescents who completed baseline surveys, most were female and categorized as older adolescents (ages 16–19 years). The majority self-identified as Black (over 90%) and reported being unemployed in both surveys. Most participants reported living in households with greater than four members (60%). The prevalence of food insecurity was high: nearly half the sample was classified as food-insecure at baseline (48%; Table 1). Although there were few statistically significant differences between adolescents classified as food-insecure versus food-secure, adolescents had a lower risk of experiencing food insecurity at baseline if they lived in a smaller household (less than four people) (*X*^2^[1] = 4.71, *p* = 0.03) and were younger than 18 (*X*^2^[1] = 3.87, *p* = 0.05). Working at least part-time (*X*^2^[1] = 2.41, *p* = 0.12), identifying as male versus female (*X*^2^[1] = 1.73, *p* = 0.19), and identifying as Black versus other race (*X*^2^[1] = 0.16, *p* = 0.69) did not have a statistically significant association with food insecurity. Participants lost to attrition were similar demographically to those who were retained at follow-up, although participants lost to attrition were slightly more likely to be classified as food-insecure and be unemployed (Supplemental Table S2). A schematic diagram with an overview of the study workflow and sampling can be found in Figure 1.

### 3.2. Participation in Federal Nutrition Assistance Programs

Participants were categorized into three groups based on food assistance status during the COVID-19 lockdown time period: 63.9% of participants (*n* = 179) received P-EBT and/or Summer SNAP in addition to traditional SNAP payments, 12.5% (*n* = 35) received only traditional SNAP payments, and 23.6% (*n* = 66) did not receive any of these three food assistance payments during the lockdown period.

**Figure 2:**
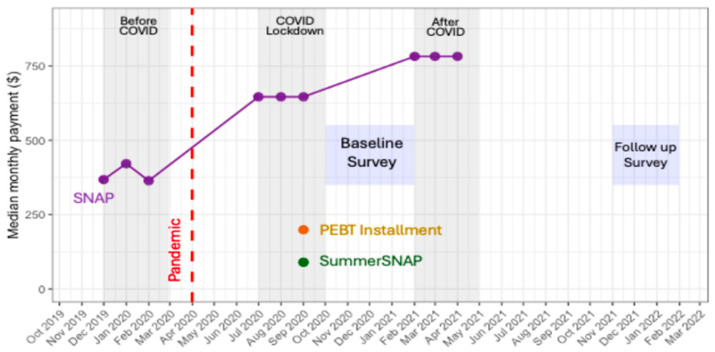
Median monthly payment (USD) from SNAP, Summer SNAP, and P-EBT. Data points are the median amounts received by all participants for each program per month.

The overall median monthly payments among all federal nutrition assistance programs increased significantly from USD 421 a month pre-pandemic to USD 646 a month pre-baseline survey and then USD 728 a month in the pre-follow-up survey (*p* < 0.01) (Figure 2). All participants lived in households that were eligible for the SNAP program at the time of the study and had participated in the program at some point. Examining the amounts by program, we observed that monthly median SNAP benefits received by households in our study increased from USD 408 prior to the COVID-19 pandemic to USD 782 in the pre-follow-up time point. Nearly 80% of the sample received a P-EBT installment of USD 199, and 38% received a one-time Summer SNAP payment of USD 90. For both P-EBT and Summer SNAP, payments were distributed to families during the lockdown time point.

### 3.3. Association between Food Security Status and Federal Assistance Programs

Table 2 displays the bivariate associations between food security status and participation in federal nutrition assistance programs. Notably, there were no statistically significant associations between adolescent food insecurity and federal nutrition assistance program status during the COVID-19 lockdown.

In the logistic regression analysis for the relationship between food insecurity and federal assistance status during the COVID-19 lockdown period, no covariates were found to be significant in the model and therefore were not used in the final model. We found that compared to those with no federal assistance, neither those who received SNAP only nor those who received SNAP with P-EBT or Summer SNAP differed significantly in risk of reported food insecurity during the lockdown period (Table 3).

For the 125 participants who completed both surveys, longitudinal analyses could be performed to compare the percentage change in participants who experienced food insecurity at each survey time point between federal assistance status groups. Due to the smaller sample size of this longitudinal sample, federal assistance status was measured by a dichotomous indicator with 63.2% (*n* = 79) categorized as receiving SNAP benefits plus P-EBT and/or Summer SNAP in the COVID-19 lockdown period and 36.8% (*n* = 46) categorized as only receiving SNAP or no benefit payments during the lockdown period. Of those who received P-EBT or Summer SNAP (*n* = 79), 46.8% reported food insecurity at baseline (approximately two months after receiving these benefits) and 39.2% at follow-up (post-lockdown), an absolute decrease of 7.6%. Of those who did not receive P-EBT or Summer SNAP (*n* = 46), 54.4% reported food insecurity at baseline and 41.3% at follow-up, an absolute decrease of 12.8%.

## 4. Discussion

This study aimed to evaluate the association between household participation in SNAP, Summer SNAP, and P-EBT and food security status among adolescents living in SNAP-eligible households in Baltimore during the COVID-19 pandemic. We did not find any significant associations between adolescent food security and participating in any of the federal nutrition assistance programs.

Our findings call into question the ability of federal nutrition assistance programs, such as SNAP or P-EBT, to influence adolescent food security. Even before the pandemic, research had indicated that SNAP benefits were largely inadequate for meeting the nutritional needs of adolescents, which are considerably higher than the needs for adults or younger children in the household [37,38]. The amount of food assistance benefits is based on the premise that families will primarily buy nutritious foods (e.g., fruits, vegetables, and meat). However, the reality is that less expensive foods are usually higher in sugar, sodium, and saturated fat and are more convenient, and nutritious options are often more expensive and require more time, knowledge, and skills to prepare [24].

Another reason that may explain why there was no association between participation in federal nutrition assistance programs and adolescent food security status is that federal benefits are received by the head of household, not the adolescent. Although this may make sense for most households in the United States where parents do the food shopping and preparation for their children [39], in many low-income households, adolescents are often responsible for feeding not only themselves but also their younger siblings [40]. Similarly, in our study, we found that larger households were associated with adolescent food insecurity. This may suggest that there was not enough food for all members of the household and that adolescents may have reduced or changed their intake to protect other members of the household. This aligns with previous research which has found that adolescents sometimes skip meals or eat less to ensure food security for other members of the household [41].

The local food environment also plays a role in how federal nutrition assistance programs may influence food security status. In Baltimore, structural racism is tied to historic policies that have led to disenfranchised neighborhoods and inequitable access to resources, such as healthy food [42]. Among Baltimore residents, African Americans disproportionately experience food insecurity and are more likely to live in ‘food deserts’ compared to other racial or ethnic groups [43]. In this sense, increasing benefits alone may not be enough to address the barriers that adolescents in low-income houses face in acquiring food, especially during economic crises.

Although receiving additional food assistance program benefits was not associated with food security status among adolescents, we still observed an increase in the prevalence of food security between survey rounds. This may be related to other programs not calculated in our analysis, such as the Child Tax Credit and/or Economic Impact Payments (colloquially: stimulus checks). Additional programs implemented in Baltimore during the pandemic may have also contributed to improving adolescent food security, such as community food pantries and increased meal distribution sites throughout the city. More research is needed to further explain the factors that influence household and adolescent food insecurity in Baltimore.

Several limitations of this study should be considered with our results. First, collecting data among adolescents during the pandemic posed several challenges. Parents and adolescents often did not answer our phone calls or our text messages, and consequently, many potential respondents were never reached. Second, several issues reduced participation in the second survey round, including participant cell phone numbers being changed or discontinued and adolescents relocating to sites outside Baltimore in between survey rounds. Also, after schools reopened, availability for recruiting and administering the survey to adolescents was diminished. Relatedly, we had to delete 65 adolescents from the analyses as we were unable to match their unique identifiers to the Maryland state database. Consequently, the sample sizes of our surveys—and particularly the second survey (*n* = 131)—were reduced, which further limited the analyses that could be performed. In addition, our measure of food insecurity was at the individual level and not at the household level. While this provides critical data on adolescents, it limits our ability to compare with studies that measure food insecurity at a household level. Finally, the present results may not be generalizable to all adolescents experiencing food insecurity in Baltimore City, as our sample was restricted to adolescents who lived in households eligible for SNAP benefits and completed an application to a summer employment program. Households in our sample, therefore, may have lower levels of income compared to households not eligible for SNAP. Additionally, given our sample included young people who applied to the YouthWorks program and were eligible to receive SNAP benefits, households in our sample may also be more connected to supports and services compared to households which never received SNAP or engaged with the YouthWorks program.

## 5. Conclusions

Despite these limitations, this study offers a unique examination of adolescent food security during the pandemic by matching Maryland’s state data on household participation in SNAP, P-EBT, and Summer SNAP before the COVID-19 pandemic and during the pandemic with survey data on food security among adolescents aged 14–19 years in Baltimore. It is now well documented that the COVID-19 pandemic magnified existing health disparities for historically oppressed populations and particularly highlighted the need for increased food assistance for low-income households. The findings from this study showed that although federal nutrition assistance programs during the pandemic resulted in families receiving more than USD 700 more a month, they were not associated with improved food security among adolescents. Although programs such as SNAP and P-EBT have been shown to be successful in reducing food insecurity among adults and younger children in the household, additional strategies may be needed to ensure that the needs of adolescent children are being met. Additional strategies may include food delivery or access programs to improve fresh food availability for adolescents and families as a supplement to financial support, and these should be evaluated in future research. Increased attention on how best to improve adolescent food security in low-income households is clearly warranted, as food insecurity among adolescents can carry long-term negative health consequences well into adulthood.

## Figures and Tables

**Table 1:** Sample characteristics of baseline survey participants (*n* = 280) by food security status.

	Total Sample (*n* = 280) ^1^	Food-Secure (*n* = 149) ^1^	Food-Insecure (*n* = 135) ^1^
**Race**			
Black	261 (93.21%)	136 (93.79%)	125 (92.59%)
Other	19 (6.79%)	9 (6.21%)	10 (7.41%)
**Gender**			
Male	88 (31.43%)	51 (35.17%)	37 (27.41%)
Female	190 (67.86%)	94 (64.83%)	96 (71.11%)
Non-binary	1 (0.36%)	0 (0%)	1 (0.74%)
My gender is not listed	1 (0.36%)	0 (0%)	1 (0.74%)
**Age**			
14–15	83 (29.64%)	50 (34.48%)	33 (24.44%)
16–17	117 (41.79%)	61 (42.07%)	56 (41.48%)
18–19	80 (28.57%)	34 (23.45%)	46 (34.07%)
**Household Size**			
<4 members of household	93 (33.21%)	56 (38.62%)	37 (27.41%)
4 members of household	59 (21.07%)	32 (22.07%)	27 (20%)
>4 members of household	128 (45.71%)	57 (39.31%)	71 (52.59%)
**Job**			
Yes, full-time (at least 40 h a week)	8 (2.86%)	3 (2.07%)	5 (3.7%)
Yes, part-time (less than 40 h a week)	49 (17.5%)	21 (14.48%)	28 (20.74%)
No	220 (78.57%)	118 (81.38%)	102 (75.56%)
Prefer not to answer	3 (1.07%)	3 (2.07%)	0 (0%)

^1^ *n* (%).

**Table 2:** Bivariate associations of food insecurity and federal assistance program participation at baseline and follow-up surveys.

The Food Assistance Program Participation Period Corresponding to COVID-19 Lockdown Includes July 2020 to October 2020						
COVID-19 Lockdown Food Assistance Category	Baseline Survey Participants (*n* = 280)			Follow-Up Survey Participants (*n* = 125)		
	Food-Secure (*n* = 145) ^1^	Food-Insecure (*n* = 135) ^1^	*p*-Value ^2^	Food-Secure (*n* = 75) ^1^	Food-Insecure (*n* = 50) ^1^	*p*-Value ^2^
No Federal Assistance	34 (51.5%)	32 (48.5%)	0.95	20 (60.6%)	13 (39.4%)	0.91
SNAP Assistance	19 (54.3%)	16 (45.7%)		7 (53.9%)	6 (46.2%)	
SNAP Assistance w/P-EBT and/or Summer SNAP	92 (51.8%)	87 (48.6%)		48 (60.8%)	31 (39.2%)	

^1^ *n* (row %). ^2^ Chi-squared test for proportions.

**Table 3:** Odds ratios for adolescent food insecurity by federal assistance program participation groups.

Federal Assistance Group	Odds Ratio (95% CI)	*p*-Value
No Federal Assistance (*n* = 66)	1.00	Reference group
SNAP Assistance Only (*n* = 35)	0.89 (0.34, 2.04)	0.79
SNAP Assistance w/P-EBT and/or Summer SNAP (*n* = 179)	1.00 (0.57, 1.77)	0.99

CI: confidence interval.

## Data Availability

Data described in the manuscript, code book, and analytic code will be made available upon request pending application and approval.

## Supplementary Materials

The following supporting information can be downloaded at: https://www.mdpi.com/article/10.3390/nu16172876/s1, Table S1: Median Monthly Payments and Participation by Time Period; Table S2: Follow Up Survey Sample Included and Lost to Follow Up. I will upload these tables.
