# Supplementary material for: The Association between Federal Nutrition Assistance Programs and Adolescent Food Security during the COVID-19 Pandemic: Evidence from Baltimore, Maryland
Source: Nutrients. 2024 Aug 28;16(17):2876. doi: 10.3390/nu16172876 (PMC11397004; doi:10.3390/nu16172876)
Supplement: Supplementary file 1 [file nutrients-16-02876-s001.zip › nutrients-3146415-supplementary.pdf]

Supplemental Table S1. Median Monthly Payments and Participation by Time Period.

	Before COVID [1]	During COVID Lockdown [1]	After COVID Lockdown [1]	*p*-Value [2]
Overall Median Monthly	$454 ($257, $646)	$646 ($509, $768)	$782 ($616, $929)	<0.05
SNAP Median Monthly	$408 ($221, $577)	$646 ($509, $768)	$782 ($616, $929)	<0.05
P-EBT Median Monthly	--	$199 ($100, $299)	--	
% Receiving P-EBT	--	172 (79.3%)	--	
Summer SNAP Median Monthly	--	$90 ($90, $90)	--	
% Receiving Summer SNAP	--	82 (37.8%)	--	

[1] Median (IQR); Median (%); [2] Kruskal-Wallis rank sum test.

Supplemental Table S2. Follow-up Survey Sample Included and Lost to Follow Up.

	**Follow-Up Survey Included Sample (N = 131)**	**Sample Lost to Follow Up (N = 149)**
**Race**		
Black	120 (91.60%)	141 (94.63%)
Other	11 (8.40%)	8 (5.37%)
**Gender**		
Male	39 (29.77%)	49 (32.89%)
Female	91 (69.47%)	99 (66.44%)
Other	1 (0.76%)	1 (0.67%)
**Age**		
14–15	48 (36.64%)	35 (23.49%)
16–17	43 (32.82%)	74 (49.66%)
18–19	40 (30.53%)	40 (26.85%)
**Household Size**		
<4 members of household	37 (28.24%)	56 (37.58%)
4 members of household	28 (21.37%)	31 (20.81%)
>4 members of household	66 (50.38%)	62 (41.61%)
**Job**		
Yes, full-time (at least 40 h a week)	3 (2.29%)	5 (3.36%)
Yes, part-time (less than 40 h a week)	23 (17.56%)	26 (17.45%)
No	102 (77.86%)	118 (79.19%)
Prefer not to answer	3 (2.29%)	0 (0.00%)
**Food Insecurity**		
Yes	65 (49.62%)	70 (46.98%)
No	66 (50.38%)	79 (53.02%)
**Benefits at Survey 2**		
SNAP and (P-EBT or Summer SNAP)	84 (64.12%)	95 (63.76%)
SNAP only	13 (9.92%)	22 (14.77%)
No Payments	34 (25.95%)	32 (21.48%)
